# Association of immune checkpoint inhibitor-induced bullous pemphigoid with underlying cancer type: A lack of association with cancer tissue *COL17A1* mutations and dysregulation

**DOI:** 10.1016/j.xjidi.2026.100450

**Published:** 2026-01-13

**Authors:** Rachel C. Chang, Henning Olbrich, Yulu F. Wang, Haley Gainer, Nina Curkovic, Theresa L. Walunas, Jessica Shiu, Parul Goyal, Adrian P. Mansini, Ralf J. Ludwig, Kyle T. Amber

**Affiliations:** 1 Department of Dermatology, Rush University Medical Center, Chicago, Illinois, USA; 2 Department of Dermatology, University of Lübeck, Lübeck, Germany; 3 Department of Dermatology, University of Texas Southwestern, Dallas, Texas, USA; 4 Division of General Internal Medicine, Department of Medicine, Northwestern University Feinberg School of Medicine, Chicago, Illinois, USA; 5 Department of Dermatology, University of California Irvine, Irvine, California, USA

**Keywords:** Bullous pemphigoid, Cancer immunotherapy, COL17A1, Immune checkpoint inhibitors, Immune-related adverse events

## Abstract

Bullous pemphigoid (BP) is an autoimmune blistering disease caused by autoantibodies to collagen type 17 (*COL17A1*) and is a recognized immune-related adverse event in patients receiving immune checkpoint inhibitors (ICIs). We investigated whether cancer type influences the risk of developing ICI-induced BP and whether *COL17A1* mutations or dysregulation in tumor tissue contributes to disease-specific variation. Using TriNetX, systematic review, and bioinformatics datasets, we comprehensively assessed the associations of ICI-induced BP with different malignancy types as well as *COL17A1* gene expression, mutation frequency, and immune correlations across cancers. Lung cancer was the most common underlying malignancy in ICI-induced BP, but nonmelanoma skin cancer and renal cell carcinoma had the highest relative risk, whereas lung cancer had the lowest. ICI-induced BP was associated with improved survival across several cancers. Urothelial cancer showed the shortest time to onset, whereas renal cell carcinoma showed the longest. Cutaneous squamous cell carcinoma and melanoma exhibited the highest *COL17A1* mutation burden, whereas renal cell carcinoma had a low burden. *COL17A1* was overexpressed in several cancers but underexpressed in melanoma, without strong correlation to tumor-infiltrating immune cells. Although the incidence of ICI-induced BP significantly differed on the basis of cancer type, *COL17A1* mutations or dysregulation do not appear to drive this phenomenon, suggesting alternative immune mechanisms.

## Introduction

Bullous pemphigoid (BP) is an autoimmune blistering skin disease that is most common in elderly individuals (Ujiie, 2023). It is characterized by the presence of autoantibodies targeting the hemidesmosomal proteins BP180 (also known as collagen type XVII) and BP230 (Cole et al, 2022), leading to linear deposition of IgG and complement along the basement membrane zone (Moro et al, 2020). Clinically, BP usually appears as generalized pruritic urticarial plaques and subepidermal tense blisters (Vaillant et al, 1998). Histopathological examination reveals subepidermal blisters with dermal inflammatory infiltration predominantly composed of eosinophils and neutrophils (Schmidt and Zillikens, 2013).

In recent years, immune checkpoint inhibitors (ICIs) have revolutionized the treatment landscape for multiple cancers (Chen et al, 2024). These mAbs block key inhibitory immune checkpoints, such as PD-1 (programmed cell death protein 1), PD-L1 (programmed death-ligand 1), and CTLA-4 (cytotoxic T-lymphocyte-associated protein 4) (Pauken et al, 2019; Wang et al, 2023), thereby enhancing antitumor T-cell responses (Hasan Ali et al, 2020). ICIs have demonstrated efficacy across a broad range of malignancies, including melanoma, nonsmall cell lung cancer, renal cell carcinoma (RCC), hepatocellular carcinoma, urothelial carcinoma, colorectal cancer, and Hodgkin lymphoma (Hussaini et al, 2021).

However, ICIs frequently induce immune-related adverse events, with cutaneous toxicities being among the most common (Asdourian et al, 2022; Postow et al, 2018; Sibaud, 2018), The clinical manifestations of cutaneous immune-related adverse events vary widely and can range from mild reactions, such as maculopapular rash and pruritus, to severe autoimmune blistering diseases, such as BP (Tan et al, 2025; Wang et al, 2023). ICI-induced BP (ICI-BP) occurs in an estimated 0.3–0.8% of patients receiving ICI therapy (Kawsar et al, 2022; Said et al, 2022; Tan et al, 2025; Ujiie, 2023). Although the precise mechanisms remain unclear, it is thought that ICIs disrupt immune tolerance, leading to autoimmune reactions against the skin (Sibaud, 2018). ICIs’ antitumor immunity activation is not tumor specific and can result in T-cell dysfunction and self-reactivity against tumor antigens (Li et al, 2024). Antigen cross-reactivity is a potential mechanism, because BP180 can be expressed on tumors and normal skin, suggesting that when ICIs activate the immune system to attack tumor cells expressing BP180, they may simultaneously target BP180 in basement membrane zone (Hasan Ali et al, 2020; Pujalte-Martin et al, 2020). In addition, ICI therapy may dysregulate B-cell function, promoting the production of pathogenic autoantibodies such as anti-BP180 IgG, which has been linked to both increased skin toxicity and favorable oncologic outcomes (Toi et al, 2019).

Given the central role of BP180 in BP pathogenesis, we hypothesized that tumor-specific alterations, such as gene mutations or dysregulated expression, might lead to cancer-type–specific differences in the incidence of ICI-BP. To test this, we conducted a multimodal study combining a retrospective population-based analysis with a systematic literature review. We further leveraged bioinformatics tools to assess *COL17A1* gene expression, mutational burden, and associations with immune infiltration across different cancers. This comprehensive approach aimed to elucidate whether tumor-intrinsic features of *COL17A1* could account for the variable risk of BP development during ICI therapy.

## Results

### Population study

**Figure 1:**
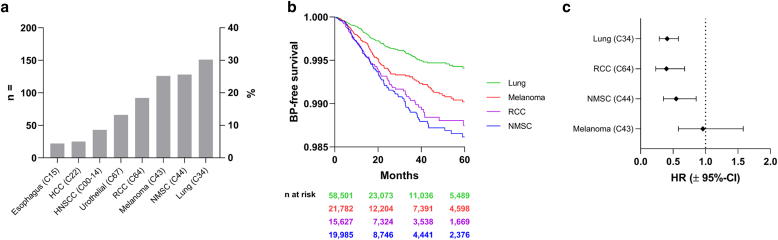
**Analysis of Trinetx database for cases of ICI-BP.** (**a**) Relative frequency of underlying cancers associated with ICI-BP. **(b)** BP-free survival, defined as time from ICI initiation to development of BP or death, demonstrates the greatest risk over time in patients with NMSC. **(c)** Hazard ratios with 95% CIs of 1:1 propensity-score–matched patients with or without ICI-BP reveal decreased mortality in all 4 major cancer subtypes. BP, bullous pemphigoid; CI, confidence interval; HCC, hepatocellular carcinoma; HNSCC, head and neck squamous cell carcinoma; ICI-BP, immune checkpoint inhibitor–induced bullous pemphigoid; HR, hazard ratio; NMSC, nonmelanoma skin cancer; RCC, renal cell carcinoma.

Using the TriNetX platform, we identified 505 cases of ICI-BP. Among these cases, lung cancer followed by melanoma was the most frequent (Figure 1a). To minimize potential bias of including patients with nonmelanoma skin cancer (NMSC) who received ICI for other indications, we performed a validation analysis by adding International Classification of Diseases, Tenth Revision, Clinical Modification (ICD-10-CM) code Z12.83 (encounter for screening for malignant neoplasm of skin) to the propensity score matrix and repeated the analysis with all ICIs for which the results were unaffected, supporting that utilization of dermatologic care did not result in selection bias. Assessing BP-free survival, that is, the probability of not developing BP, revealed that NMSC followed by RCC had the greatest risk of developing ICI-BP, whereas lung cancer had the lowest risk (Figure 1b). Five-year survival among patients with ICI-BP versus patients receiving ICI without BP for each respective cancer except melanoma revealed a significant risk reduction in all-cause mortality (Table 1 and Figure 1c).

**Table 1:** Hazard Ratios for Mortality in 1:1 Propensity Score–Matched Patients with or without ICI-BP Reveals Decreased Death in Patients with ICI-BP

Cancer	Cases *n*	Cases Outcomes	Controls *n*	Controls Outcomes	*P*-Value	HR	HR 95% CI	
Lung (C34)	151	52	150	78	<.001	0.409	0.287	0.583
Melanoma (C43)	125	36	123	27	.87	0.959	0.582	1.581
Kidney (C64)	92	25	91	32	<.001	0.398	0.233	0.678
Nonmelanoma skin (C44)	133	36	133	43	.007	0.548	0.351	0.855

Abbreviations: CI, confidence interval; HR, hazard ratio; ICI-BP, immune checkpoint inhibitor–induced bullous pemphigoid.

### Systematic review of clinical trials

To evaluate the incidence of ICI-BP in randomized controlled trials, we analyzed data from a previously published systematic review (Curkovic et al, 2024). Among 46,134 patients, only 8 patients were reported to develop BP. However, most trials did not describe workup or morphology of dermatitis, presumably underdiagnosing BP. Furthermore, diagnostic criteria for BP were not standardized across studies. Given these limitations, we concluded that data from clinical trials lacked the resolution required for reliable analysis and instead pursued a systematic review of published cases to obtain detailed patient-level information.

### Systematic review of cases

**Figure 2:**
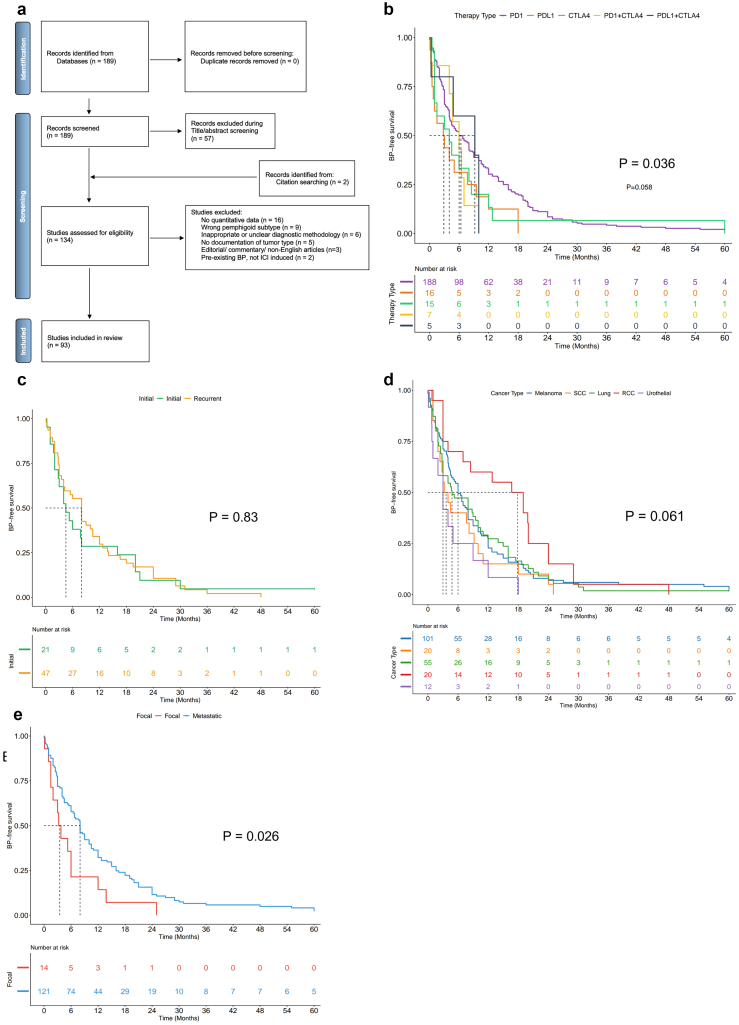
**Systematic review and analysis of ICI-BP latency based on cancer type.** (**a**) PRISMA flow chart demonstrating article selection strategy. **(b)** Kaplan–Meier curve demonstrating BP-free survival on the basis of cancer subtype. (**c–e**) Kaplan–Meier curve comparing BP-free survival between **(c)** ICI treatment for initial and recurrent tumors, **(d)** various ICI therapy mechanisms, or **(e)** focal and metastatic disease. *P*-values reflect (**b, c, e**) Cox proportion hazards or (**d**) log-rank test. ICI-BP, immune checkpoint inhibitor–induced bullous pemphigoid; NSCLC, nonsmall cell lung cancer; PRISMA, Preferred Reporting Items for Systematic Reviews and Meta-analyses; RCC, renal cell carcinoma; SCC, squamous cell carcinoma.

We next conducted a systematic review of published case reports and retrospective case series using standardized diagnostic criteria for BP, including clinical presentation, direct immunofluorescence, and either salt-split skin indirect immunofluorescence or BP180/BP230 ELISA positivity. The PRISMA (Preferred Reporting Items for Systematic Reviews and Meta-analyses) flow chart is shown in Figure 2a. We identified a total of 278 cases of ICI-BP. Patient demographics are reported in Table 2.

**Table 2:** Demographics, Treatment, and Outcomes of Patients from Systematic Review of ICI-BP

Characteristics	Patient Count (%)
Total patients	278 (100)
Sex	
Male	169 (60.8)
Female	55 (19.8)
Unknown	54 (19.4)
Cancer type	
Melanoma	118 (42.4)
Lung	66 (23.7)
RCC	24 (8.6)
SCC	20 (7.2)
Urothelial	14 (5.0)
HNSCC	9 (3.2)
Skin	5 (1.8)
Endometrial	2 (0.7)
Merkel	2 (0.7)
Colorectal	2 (0.7)
Breast	2 (0.7)
Other	14 (5.0)
Focal/metastatic	
Focal	14 (5.0)
Metastatic	136 (48.9)
Unknown	128 (46.0)
Initial/recurrent	
Initial	22 (7.9)
Recurrent	51 (18.3)
Unknown	205 (73.7)
Nodal metastasis	
Yes	34 (12.5)
No	14 (5.2)
Unknown	230 (82.3)
Treatment type	
PD-1	192 (69.1)
PD-L1	17 (6.1)
CTLA4	19 (6.8)
PD-1 + CTLA4	7 (2.5)
PD-L1 + CTLA4	5 (1.8)
Unknown	38 (13.7)
PD-1/PD-L1 drug	
Pembrolizumab	102 (36.7)
Nivolumab	97 (34.9)
Atezolizumab	8 (2.9)
Durvalumab	4 (1.4)
Cemiplimab	1 (0.4)
Sintilimab	1 (0.4)
Unknown	65 (23.3)
Treatment outcome	
Stopped	130 (46.8)
Continued	50 (18.0)
Death	18 (6.5)
Unknown	80 (28.8)
Bullous pemphigus Management	
Oral steroids	198 (71.2)
Topical steroids	193 (69.4)
Bullous pemphigus outcome	
Resolved	107 (38.5)
Persistent	31 (12.5)
Partial	36 (12.9)

Abbreviations: HNSCC, head and neck squamous cell carcinoma; ICI-BP, immune checkpoint inhibitor–induced bullous pemphigoid; RCC, renal cell carcinoma; SCC, squamous cell carcinoma.

Time from ICI initiation to onset of BP symptoms was reported in 238 cases. We thus utilized this delay from treatment initiation to perform Kaplan–Meier analyses to better understand temporal associations. Cancer type was significantly associated with BP-free survival, with urothelial cancer associated with the fastest onset of BP cases, and RCC demonstrating a more delayed onset (*P* = .036) (Figure 2b). Neither tumor recurrence (*P* = .825) nor ICI mechanism of action (*P* = .058) significantly affected BP-free survival (Figure 2c and d). However, patients with localized disease at treatment initiation exhibited significantly shorter BP-free survival than those with metastatic disease (*P* = .026) (Figure 2e).

We next assessed BP treatment and outcome in patients with BP-ICI. Notably, most patients received oral corticosteroids, and 38.5% achieved complete BP resolution. Further outcome analyses were limited by the absence of control groups. Outcome data are shown in Table 2.

### Analyzing *COL17A1* mutations in cancer

**Figure 3:**
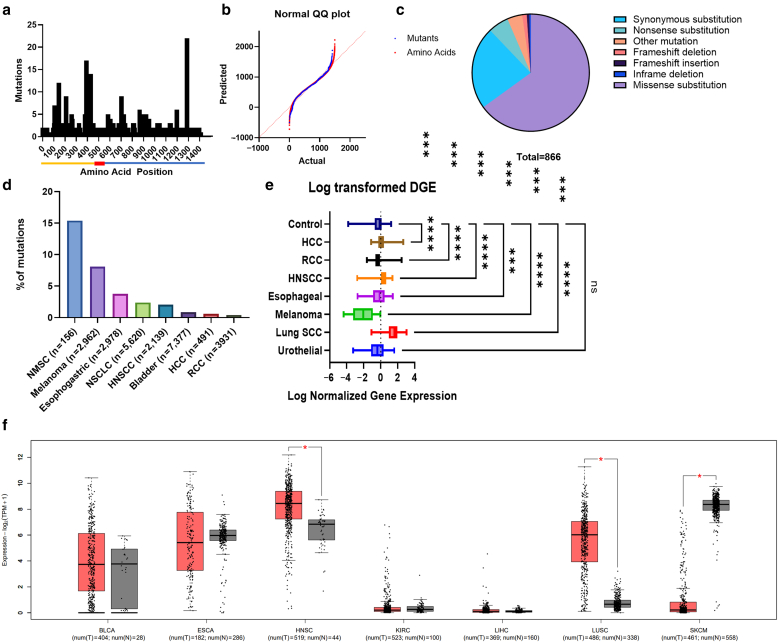
**Mutations and differential gene expression of *COL17A1* in cancer.** (**a**) Mapping of *COL17A1* mutants to the BP180 protein. Intracellular, NC16a, and extracellular domains are shown in yellow, red, and blue, respectively. **(b)** QQ plot demonstrates distinct distribution of mutations relative to amino acid (*P* < .001) of full-length protein with lack of mutations along the distal C-terminus. **(c)** Percentage of tumors assessed carrying *COL17A1* mutation. **(d)** Mutation burden in *COL17A1* across tumor types. **(e)** Box plot (minimum to maximum) of log-transformed tumoral differential gene expression for each tumor type relative to its normal tissue control (1-way ANOVA with Dunnett’s posthoc test). **(f)** Box plot of log_2_(TPM+1) transformed tumor versus control tissue gene expression (1-way ANOVA). ∗*P* < .05 and ∗∗∗*P* < .001. BLCA, bladder urothelial carcinoma; ESCA, esophageal carcinoma; HCC, hepatocellular carcinoma; HNSCC, head and neck squamous cell carcinoma; KIRC, kidney renal clear cell carcinoma; LIHC, liver hepatocellular carcinoma; LUSC, lung squamous cell carcinoma; NMSC, nonmelanoma skin cancer; NSCLC, nonsmall cell lung cancer; RCC, renal cell carcinoma; SCC squamous cell carcinoma; SKCM, skin cutaneous melanoma; TPM, transcripts per million.

Given the observed differences of ICI-BP between certain types of cancers, we next examined whether the mutational burden of the primary autoantigen in BP, *COL17A1*, differed among cancers. We first performed a mapping of *COL17A1* gene mutations across cancer in various tissue compartments using the COSMIC (Catalogue of Somatic Mutations in Cancer) database. We analyzed 49,382 cancer genomes and mapped respective mutations to the corresponding protein sequence. This revealed sparing of the C-terminal ectodomain but otherwise a normalized distribution without clustering across any 1 domain of BP180 (Figure 3a and b). Of mutations, missense mutations were the most frequent, followed by synonymous substitutions (Figure 3c). We then bioinformatically evaluated mutational burden in individual cancer types using the cBioPortal. This revealed the highest mutation burden in NMSC followed by melanoma. In contrast, RCC exhibited a markedly low mutational burden (Figure 3d)

### Assessing *COL17A1* gene expression in cancer

**Figure 4:**
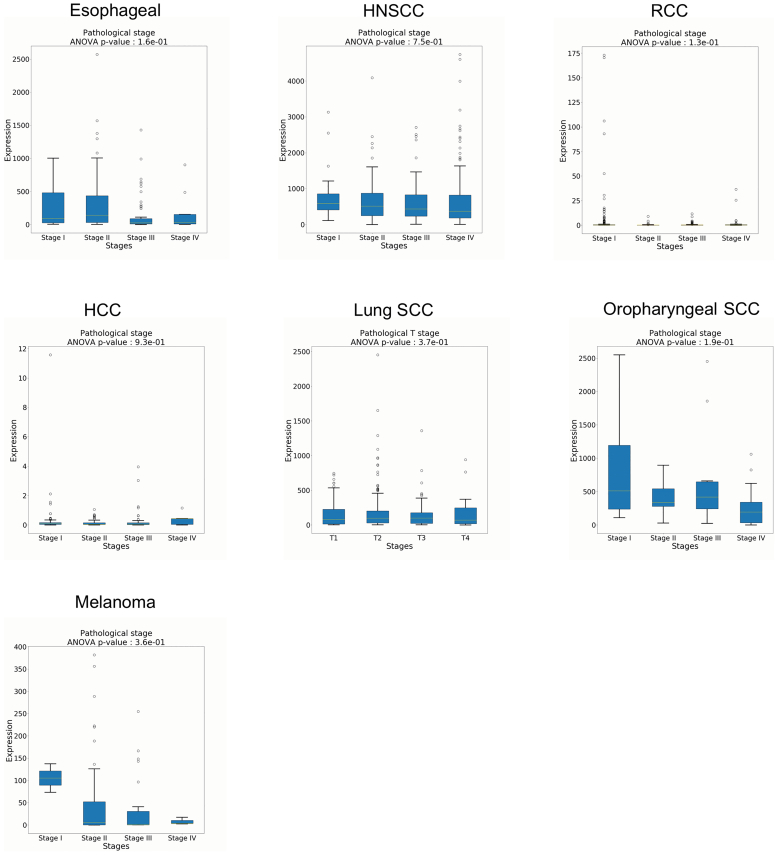
**Box plots of COL17A1 expression stratified by tumor stage demonstrates that COL17A1 expression does not significantly vary across tumor stages.** HCC, hepatocellular carcinoma; HNSCC, head and neck squamous cell carcinoma; RCC, renal cell carcinoma; SCC, squamous cell carcinoma.

**Figure 5:**
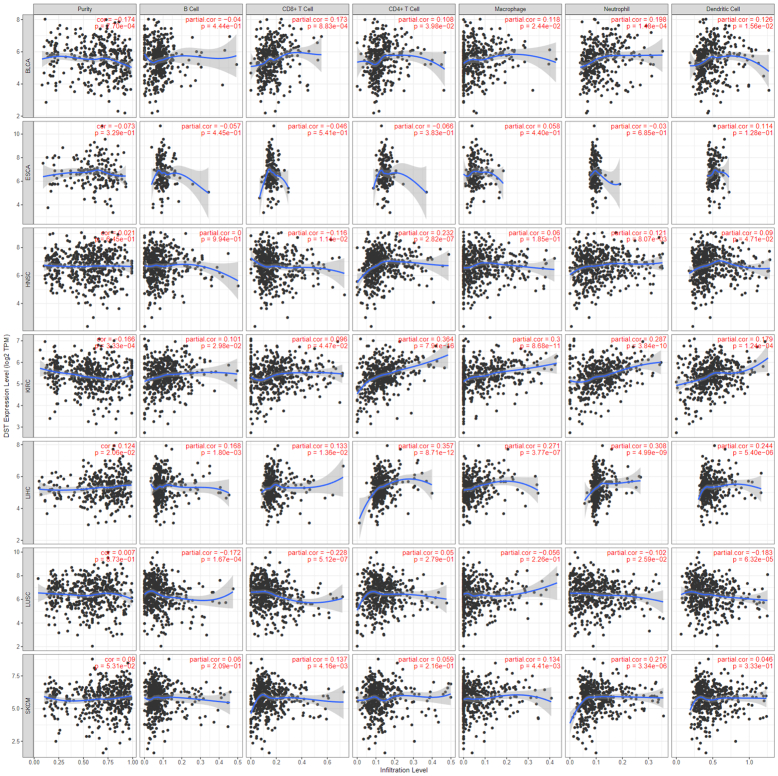
**Spearman correlation plots of *COL17A1* expression relative to immune cell tumoral infiltration.** HCC, hepatocellular carcinoma; HNSCC, head and neck squamous cell carcinoma; RCC, renal cell carcinoma; SCC, squamous cell carcinoma.

We next assessed relative gene expression of *COL17A1* in cancer types matched to paired normal tissue using the OncoDB database. This revealed that *COL17A1* was upregulated in lung squamous cell carcinoma (SCC), esophageal SCC, head and neck SCC, RCC, and hepatocellular carcinoma but notably downregulated in melanoma (Figure 3e). Interestingly, dysregulation of *COL17A1* was not associated with increased tumor staging (Figure 4). Furthermore, the expression of COL17A1 did not demonstrate robust correlations with immune cell infiltration, with only very weak or weak correlations (Figure 5). Additional validation of gene expression findings was performed in a separate cohort using GEPIA (Gene Expression Profiling Interactive Analysis), confirming the downregulation of *COL17A1* in melanoma as well as upregulation in head and neck SCC and lung SCC (Figure 3f).

**Figure 6:**
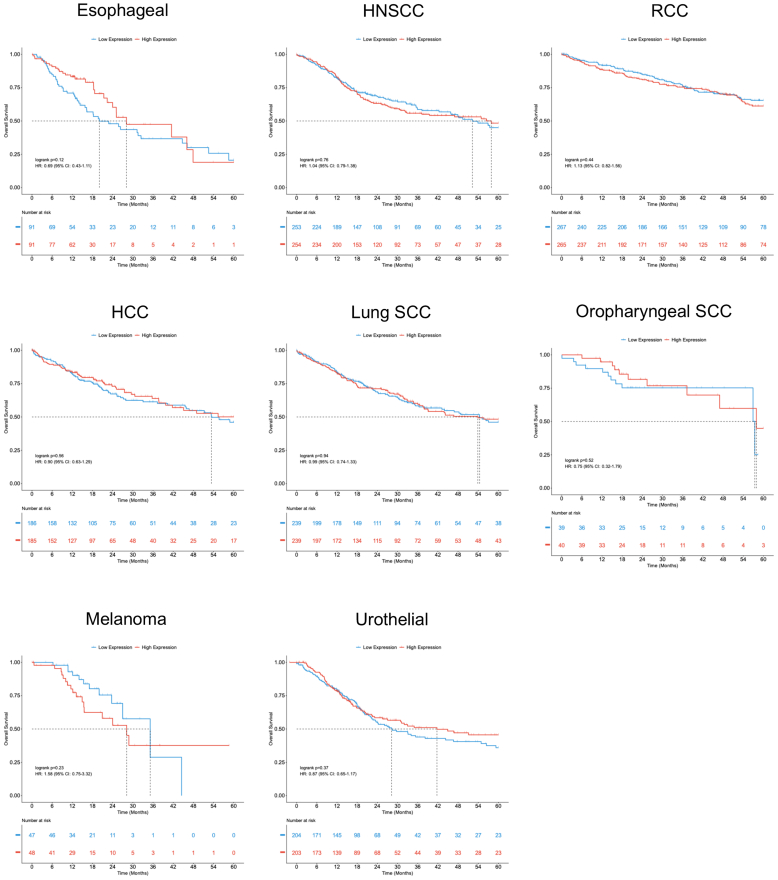
**High versus low expression of *COL17A1* is not significantly associated with survival across major tumor subtypes.** HCC, hepatocellular carcinoma; HNSCC, head and neck squamous cell carcinoma; RCC, renal cell carcinoma; SCC, squamous cell carcinoma.

**Figure 7:**
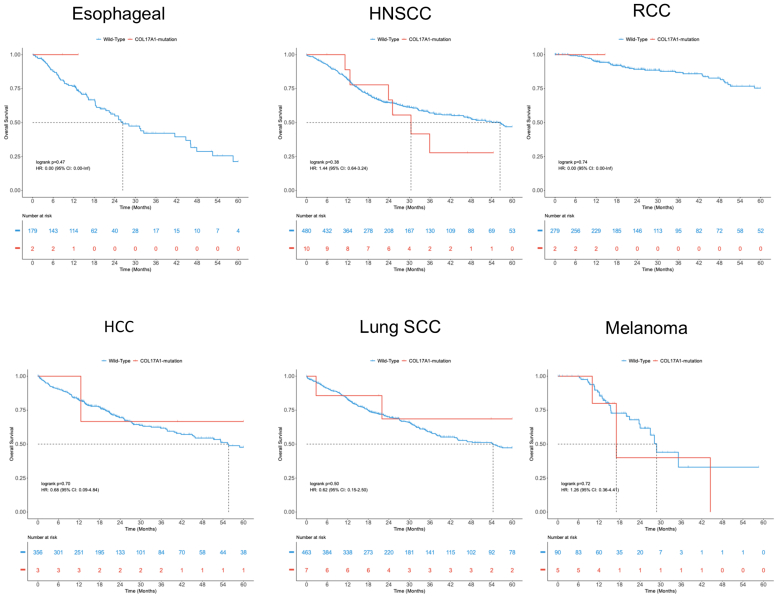
**The presence of *COL17A1* mutations does not significantly alter survival across major tumor subtypes.** HCC, hepatocellular carcinoma; HNSCC, head and neck squamous cell carcinoma; RCC, renal cell carcinoma; SCC, squamous cell carcinoma.

Finally, we assessed whether COL17A1 expression or mutational burden influenced overall cancer survival. Neither showed a significant impact across cancer types, although interpretation of mutational data was limited by small sample sizes (Figures 6 and 7). The OncoDB dataset does not include clinical data on ICI-BP development; therefore, these analyses do not test a direct relationship between *COL17A1* and ICI-BP incidence.

## Discussion

**Figure 8:**
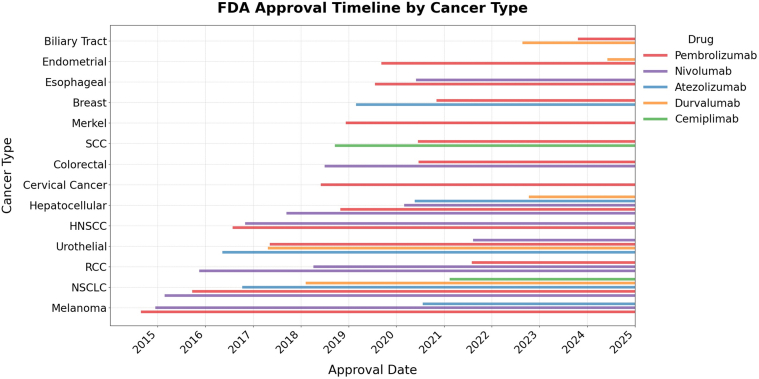
**Timeline of United States Food and Drug Administration approvals of PD-1 or PD-1L therapies for each major cancer type.** HNSCC, head and neck squamous cell carcinoma; NSCLC, nonsmall cell lung cancer; RCC, renal cell carcinoma; SCC, squamous cell carcinoma.

Our population study demonstrated that patients with cutaneous SCC and RCC had the highest risk of developing ICI-BP. Although the overall prevalence of ICI-BP in our population study was highest in lung cancer, this likely reflects the high frequency of lung cancer cases treated with ICIs relative to other tumor type. The annual incidence of nonsmall cell lung cancer was 40.9 per 100,000 persons in 2017 (Ganti et al, 2021), compared with that of RCC at an incidence rate of 22.03 per 100,000 for men and 11.4 per 100,000 for women from 2000 to 2019 (Rose and Kim, 2024). Nonsmall cell lung cancer is among the most frequently treated cancers with ICIs (Mamdani et al, 2022), thereby increasing the at-risk population for immune-related adverse events such as BP. In contrast, ICI therapy in RCC is generally reserved for advanced or metastatic cases (Ozay et al, 2025). Furthermore, Food and Drug Administration approval for ICI use in lung cancer preceded that of RCC by 5 years, which may have also contributed to this prevalence difference (Choueiri et al, 2021; Sul et al, 2016). Interestingly, findings from our population study contrast with prior literature, where melanoma is the most frequently reported cancer type associated with ICI-BP (Kawsar et al, 2022; Said et al, 2022). This discrepancy may be due to earlier approval of ICIs in melanoma (Figure 8), potential publication bias, or actual variations from previously published cohorts.

In our population study, ICI-BP was associated with significantly decreased 5-year all-cause mortality across cancer types, suggesting that it may serve as a favorable prognostic marker (Adhikari et al, 2024; Lin et al, 2022; Nieder et al, 2025; Paulson et al, 2025). This finding likely reflects a more robust immune response induced by ICI in these patients and confirms findings of improved survival after cutaneous immune-related adverse events under immunotherapy in large observational studies (Tang et al, 2022b; Zhang et al, 2023). Whether the T helper 2 inflammatory profile of BP is a sequela of robust ICI-induced response or a direct cause of an improved tumor response remains to be determined (Pruessmann et al, 2025; Sadik et al, 2020; Said et al, 2022). For example, eosinophilia is a well-characterized laboratory finding in BP, whereas the presence of eosinophilia in melanoma has, for example, been shown to portend a favorable prognosis (Ammann et al, 2022; Jones et al, 2021; Simon et al, 2020). Our systematic review provided patient-level data in a distinct subset, demonstrating further differences based on underlying cancer, including the association of urothelial cancer with the fastest onset of BP, and RCC demonstrating a more delayed onset.

Interestingly, our gene expression database analysis did not demonstrate an association between differential gene expression of *COL17A1* or mutational burden with survival. Whereas cutaneous SCC exhibited a relatively high frequency of *COL17A1* mutations (Jones et al, 2020), which could theoretically increase neoantigen formation and risk of autoimmunity, RCC had a very low mutation burden yet also showed a high ICI-BP risk (Hakimi et al, 2020). In addition, *COL17A1* expression patterns varied across tumor types; it was significantly downregulated in melanoma but elevated in urothelial cancers. These findings suggest that *COL17A1* expression or mutation alone is unlikely to be the primary driver of ICI-BP susceptibility.

BP has been highly associated with the HLA-DQB1∗03:01 allele (Amber et al, 2017). Patients with this allele show significantly increased risks of ICI-induced colitis and worse progression-free survival than patients with other alleles in urothelial cancer (Hasan Ali et al, 2019; Takahashi et al, 2022). Likewise, the presence of the DQB1∗03:01 allele in patients with melanoma was associated with advanced disease (Lee et al, 1996). A small study demonstrated that all 7 patients with ICI-BP carried the HLA-DQB1∗03:01 allele (Gandarillas et al, 2025). Thus, it remains unclear why exactly the incidence of ICI-BP differs among cancer subtypes in the absence of more robust HLA typing.

Our study has several notable limitations. TriNetX utilizes diagnostic codes and censors data for events occurring in subgroups with fewer than 10 patients, limiting the granularity of certain analyses. To mitigate misclassification, we implemented strategies validated in prior studies to ensure accurate identification of BP cases (Kridin et al, 2024, 2022). Although we aimed to determine the incidence of ICI-BP through clinical trials, having the benefit of a control population, reporting of dermatologic adverse events was sparse. Diagnostic criteria for BP were absent, and several cases of bullous eruptions lacked further detail. Therefore, we supplemented our findings with a systematic review of published cases, which provided detailed patient-level data allowing for BP-free survival analysis. However, this approach could only estimate the frequency of underlying cancer types in ICI-BP, not true incidence rates.

Despite limitations inherent to each individual data source, our combined methodological approach mitigated many of these weaknesses. We further validated findings using publicly available gene expression and mutational databases, although these resources lack individual-level patient data. Future studies comparing tumors and HLA genotypes of patients who develop ICI-BP with those of patients who do not will be critical to validating these findings.

In conclusion, we demonstrate that although most cases of ICI-BP occur in lung cancer and melanoma, the highest relative risk of developing ICI-BP is observed in those with NMSC and RCC. Cancer type was also associated with the speed of onset of ICI-BP. ICI-BP was associated with decreased death. However, neither *COL17A1* expression nor mutational burden appears sufficient to explain cancer-type–specific risk of ICI-BP, pointing toward a multifactorial etiology that likely includes HLA genotype and immune landscape.

## Materials and Methods

### TriNetX

The United States collaborative network of TriNetX was queried in November 2025 similarly to previously published protocols (Ludwig et al, 2025). The network was chosen for its high degree of covariate registration and the high number of 121 million electronic health records from 71 healthcare operations in the United States at the time of analysis. Only datasets registered within the last 20 years were included.

We first identified patients with ICI-BP by diagnosis of BP (ICD10-CM code L12.0) and preceding administration of PD-1, PD-L1, or CTLA-4 inhibitors. The ICIs considered were atezolizumab (RXNORM: 1792776), avelumab (RXNORM: 1875534), cemiplimab (RXNORM: 2058826), dostarlimab (RXNORM: 2539967), durvalumab (RXNORM: 1919503), nivolumab (RXNORM: 1597876), pembrolizumab (RXNORM: 1547545), ipilimumab (RXNORM: 1094833), and tremelimumab (RXNORM: 2619313). The cohort was then analyzed for cancer diagnoses by International Classification of Diseases, Tenth Revision codes with a look-back period of 5 years before diagnosis of ICI-BP. The 4 cancer types most prevalent in patients with ICI-BP were considered for further analysis: lung cancer (ICD-10-CM code C34), melanoma (ICD-10-CM code C43), NMSC (ICD-10-CM code C44), and RCC (ICD-10-CM code C64). Cancer survival was defined as time from ICI initiation to death, whereas BP-free survival was defined as time from ICI initiation to BP development.

Patients with a diagnosis of each cancer type and subsequent ICI administration were identified and split into cohorts with and without ICI-BP. ICI-BP cohorts and respective controls were subjected to propensity-score matching to control for relevant bias. Propensity-score matching was performed in a 1:1 ratio using the greedy nearest-neighbor approach with a caliper distance of 0.1 pooled SDs considering logit ranks derived from logistic regression. Covariates included in the regression matrix were demographic parameters; major comorbidities; administration of topical and systemic steroids’ and medication with doxycycline, mycophenolate mofetil, and dipeptidyl peptidase 4 inhibitors (Patel et al, 2021). Baseline characteristics were analyzed before and after matching. Analysis outputs are transmitted in an aggregated and deidentified form; thus, no institutional board review was necessitated prior to data collection. The deidentification process adheres to the standard defined in section §164.514(a) of the United States Health Insurance Portability and Accountability Act Privacy Rule and was formally attested to in December of 2020 by a qualified expert as specified in section §164.514(b)(1). Study results are reported according to the Strengthening the Reporting of Observational Studies in Epidemiology guidelines.

### TriNetX statistical analysis

All analyses were performed with the integrated software of TriNetX that uses R (version 4.3.1, R Foundation for Statistical Foundation, Vienna, Austria). Continuous variables were compared using *t*-tests, and categorical variables were compared using chi-square tests. Time zero (T_0_) was defined as the date of ICI initiation. Kaplan–Meier analyses were used to estimate BP-free survival and overall cancer survival after initiation of ICI, with comparisons made using the log-rank test. *P* < .05 was considered significant. Univariable Cox regression was used to express hazard ratios with 95% confidence intervals. The proportional hazards assumption was assessed statistically using the cox.zph() function on the basis of Schoenfeld residuals. Because TriNetX does not support time-varying covariate analysis, ICI-BP status was modeled as a fixed covariate. Next, to evaluate differential risks of novel ICI-BP between cancer cohorts, we similarly performed propensity-score–matched analyses comparing patients from each cancer type cohort without prevalent BP with all other patients with cancer combined. Patients were censored after their last available record in the database.

### Systematic review

This systematic review was registered at the International Prospective Register of Systematic Reviews (PROSPERO registration number CRD420251134842), was conducted following the Cochrane Collaboration’s guidelines, and reported according to the PRISMA reporting guidelines (Page et al, 2021). The PubMed electronic database was systematically searched on August 30, 2025, using a comprehensive search strategy (atezolizumab OR avelumab OR cemiplimab OR dostarlimab OR durvalumab OR ipilimumab nivolumab OR pembrolizumab OR checkpoint OR ICI OR immune checkpoint) AND (bullous pemphigoid OR pemphigoid OR pemphigoides). There were no filters or date limits. Duplicates were removed using the Bramer deduplication method (Bramer et al, 2016).

We included articles that met the following criteria: studies describing individual patients who developed BP in association with ICIs; documentation of the underlying tumor type; involvement of an ICI agent; and use of appropriate diagnostic methodology to confirm BP, such as histopathology, direct or indirect immunofluorescence, or ELISA for BP180 or BP230. We excluded studies if they described mucous membrane pemphigoid or other non-BP blistering disorders; involved pre-existing BP not induced by ICI therapy; lacked documentation of the primary tumor type; did not use or report appropriate diagnostic methodology; or were non-English articles, conference abstracts, editorials, or commentaries.

Two authors (RCC and HG) independently screened titles and abstracts and then full text of articles. Conflicting decisions were resolved by consensus with third author (KTA). Google Sheets was used for data extraction after full-text review of included article. A total of 226 studies were screened and assessed for eligibility. After applying the inclusion and exclusion criteria, 109 studies were selected for inclusion. Of the 109 studies included, 106 were case reports or case series, and 3 were retrospective cohort studies. A total of 278 cases of ICI-BP were identified. Because we utilized our systematic review to generate a retrospective cohort of patients rather than to synthesize large-scale outcomes data, risk of bias assessment and grading of evidence was not performed.

### Systematic review statistics

Data from the patient cohort were used to generate Kaplan–Meier survival plots with log-rank test performed. Proportional hazards assumption for all data was assessed using the cox.zph() function, which tests the correlation between Schoenfeld residuals and time. Where proportional hazards assumptions were met, Cox proportional hazards models were used. Survival data based on ICI therapy type, initial versus recurrent tumor, cancer type, and focal versus metastatic disease were assessed. All statistics were performed in R (version 4.3.1, R Foundation for Statistical Foundation). Statistical significance was defined as 2-sided *P* < .05.

### Mutation data

Mutations in *COL17A1* were analyzed using the COSMIC database (Sondka et al, 2024). Point mutations were categorized by type and mapped to the BP180 protein structure. Frequency of mutations relative to the expected normalized distribution was assessed using the Kolmogorov–Smirnov test. Because COSMIC primarily demonstrates mutational burden by tissue location rather than tumor subtype, we next utilized the cBioPortal (Gao et al, 2013). We focused our analysis on primary tumors for which ICI is typically utilized: melanoma, cutaneous SCC or NMSC, lung SCC, head and neck SCC, RCC, and urothelial carcinoma. We assessed the full tumor database of over 100,000 tumors because several included studies analyzed multiple cancer types outside of the scope of our analysis. We then manually selected on the basis of tumor type, corresponding to our inclusion criteria mentioned earlier.

### Gene expression and immune cell infiltration data

Gene expression data were next obtained using the OncoDB (Tang et al, 2022a), with normal tissue defined as nonmalignant tissue from the same organ as the primary cancer type in the same individual. The ratio of *COL17A1* expression to matched normal tissue expression of *COL17A1* was subsequently log transformed. One-way ANOVA with Dunnett’s posthoc test was performed to assess dysregulation of each cancer relative control tissue. Gene expression was validated using the GEPIA server (Tang et al, 2017). Survival relative to high or low expression was assessed using the OncoDB server using Kaplan–Meier curves with the log-rank test. Spearman correlations between *COL17A1* expression and immune infiltration (T cells, B cells, macrophages) were evaluated using the TIMER (Tumor Immune Estimation Resource) (Li et al, 2017).

## Ethics Statement

This study utilized deidentified and anonymous electronic health record data obtained from the TriNetX Research Network. TriNetX aggregates data from participating healthcare organizations and provides access to deidentified datasets for research purposes in compliance with the Health Insurance Portability and Accountability Act. Because all data are fully deidentified and no protected health information was accessed, this study did not require institutional review board approval or informed consent. Data access and use were conducted in accordance with TriNetX data use agreements.

## Data Availability Statement

The datasets generated and/or analyzed in the study are available from the corresponding author on request. The data analyzed in this study are available through the TriNetX Research Network (https://trinetx.com/real-world-data) to qualified researchers subject to data-use agreements. Aggregated and deidentified data were used, and no permissions were required to reuse these datasets.

## ORCIDs

Rachel C. Chang: http://orcid.org/0000-0003-4963-6926

Henning Olbrich: http://orcid.org/0000-0003-0863-7148

Theresa L. Walunas: http://orcid.org/0000-0002-7653-3650

Jessica Shiu: http://orcid.org/0000-0002-2264-8372

Adrian P. Mansini: http://orcid.org/0000-0002-0857-0556

Ralf J. Ludwig: http://orcid.org/0000-0002-1394-1737

Kyle T. Amber: http://orcid.org/0000-0002-2906-2454

## Conflict of Interest

HO and RJL received travel grants from TriNetX. The remaining authors state no conflict of interest.
